# The Combination of Different Ergonomic Supports during Dental Procedures Reduces the Muscle Activity of the Neck and Shoulder

**DOI:** 10.3390/jcm8081230

**Published:** 2019-08-15

**Authors:** José A. García-Vidal, Manuel López-Nicolás, Ana C. Sánchez-Sobrado, María P. Escolar-Reina, Francesc Medina-Mirapeix, Roberto Bernabeu-Mora

**Affiliations:** 1 Departament of Physiotherapy, University of Murcia, Campus de Espinardo, 30100 Murcia, Spain; 2 Research group Fisioterapia y Discapacidad, Institute of Biomedical Research (IMIB)-Virgen de la Arrixaca University Clinical Hospital, El Palmar, 30120 Murcia, Spain; 3 Department of Dermatology, Stomatology, Radiology and Physical Medicine, University of Murcia, 30008 Murcia, Spain; 4 Division of Pneumology, Morales Meseguer University Clinical Hospital, 30007 Murcia, Spain

**Keywords:** ergonomics, dentists, electromyography, muscle activity

## Abstract

Ergonomic supports have become popular for the prevention of musculoskeletal disorders. This study sought to evaluate the efficacy of different ergonomic supports and their combination to reduce muscle activity of the neck and shoulder muscles. A one-way repeated measures design was used to evaluate 36 practicing dentists while they performed three posterior composite restoration procedures on a phantom head. Portable surface electromyography (sEMG) recordings were used to measure the muscle activity of three muscles (Upper Trapezius, Lateral Deltoid and Anterior Deltoid) in the dominant upper extremity, with and without the use of different ergonomic supports (ergonomic stool, magnification lenses and both) during the performance of these tasks. A repeated measures analysis of variance was used. The muscle activity of all muscles differed significantly across the four ergonomic conditions during the three tasks. The use of ergonomic supports such as magnification lenses, the ergonomic stool, or the combination of both, is effective for decreasing the muscle activity of the three muscles during the three tasks, when compared to standard practice. In addition, the decrease of muscle activity was higher using magnification lenses when compared to the ergonomic stool. Furthermore, the greatest decrease was found with the combination of both supports.

## 1. Introduction

According to earlier studies, over 60% of dentists experience different types of musculoskeletal disorders (MSDs) during their professional life, of these, the most prevalent pain regions are the neck, shoulder and back [1]. The development of these MSDs may be related to strained posture, prolonged and repetitive movements, unhealthy postures and high levels of muscle activity [2,3,4,5,6].

Many ergonomic and preventive strategies have been recommended to avoid these common MSDs, such as: reducing work time and fatigue [7]; limiting the workspace [8]; alternating between standing and sitting time [9]; working with dental assistants [10]; increasing physical activity and stretching [10,11]; and the use of different ergonomic supports, such as the ergonomic stool and magnification lenses [12,13,14,15]. 

The increasingly frequent use of ergonomic supports to improve the dental workplace has led to the emergence of studies that demonstrate the utility of ergonomic stools with lumbar support [13,14,16] or magnification lenses [12,15,17,18,19]. Multiple outcome measures have been used, highlighting pain scales and functional assessments (Quebec or DASH) [19], shoulder/cervical ROM [18,19], fatigue perception [20,21], motion analysis [15,22,23], postural assessment [13,15,17,24] and muscle activity [14,15,16].

Surface electromyography (sEMG), has proven to be a valid and effective tool for the measurement of muscle activation, which has been used successfully in pathologies such as urinary incontinence [25], spasticity [26] or sports injuries [27]. Until now, sEMG has been extensively used to describe the muscle activation of dentists in standard clinical practice situations [1,20,28,29,30]. However, very few studies have used the sEMG as an outcome measure to analyze the effect of ergonomic supports such as ergonomic stools [13,14,16], and, according to our review, none have analyzed the effect of magnification lenses. Consequently, to our knowledge, no studies have compared the effect of magnification lenses and ergonomic stools, either separately or combined, on muscle activation patterns of the neck and shoulders during different dental tasks. 

In the present study, we evaluated the influence of different types of ergonomic supports (ergonomic stool and magnification lenses) on the sEMG muscle activity of three muscles of the dominant upper extremity during a posterior composite restoration procedure. Similar to the study by Finsen et al. (1998), and to enable a better analysis of the data, we divided this common dental procedure into three tasks: drilling, filling and polishing [6]. Based on earlier studies [13,14,16] our hypotheses were: a) the application of any ergonomic support during these dental procedures will reduce the degree of muscle activation compared to standard practice without ergonomic support; b) the combination of two ergonomic supports (e.g., magnification lenses and ergonomic stool) will reduce the muscle activity of all muscle groups to a greater degree when compared to the individual use of these supports.

## 2. Materials and Methods

### 2.1. Study Design

This study was based on a one-way repeated measures design. A portable surface EMG recorder was used to measure the muscle activity of three muscles from the dominant upper extremity while participants performed the three tasks of a posterior composite restoration on a phantom head: drilling, filling, and polishing. All participants performed these tasks, first without ergonomic support (baseline measures) and then using each one of three ergonomic supports with 15 min of rest time between them. Both the order in which these different ergonomic supports were used and the order in which each of the tasks were executed was randomized. In order to examine the reliability of sEMG measures during each task, we also used a test-retest design for the baseline condition using a subsample of participants, who completed two sessions 30 min apart.

### 2.2. Participants

Thirty-six dentists were recruited (*n* = 36) from among post-graduate students and professors at the University of Murcia. Eligible participants were aged 60 years or younger and without self-reported health MSDs in the back, neck and upper limbs. The exclusion criteria were visual acuity problems, previous surgery, medication that may affect the eyes, or the inability to wear glasses/prisms. All participants provided written informed consent. The study obtained approval from the Ethics Committee of the University of Murcia (approval No. 986/2014). 

### 2.3. Instrumentation

**Figure 1:**
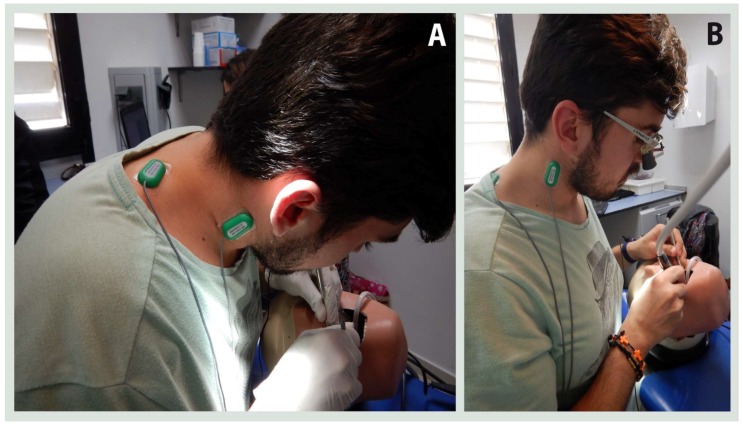
(**A**) Dentist’s posture without ergonomic support; (**B**) Dentist’s posture using magnification lenses.

All the dental procedures (and associated sEMG measures) were performed on a simulated dental workstation, which consisted of a dental chair with an adjustable height, an operating lamp Kavo Primus 2058^®^ (Kavo Dental GmbH, Biberach, Germany), a phantom head and dental model Kavo G-50 Jaw simulator^®^ (Kavo Dental GmbH, Biberach, Germany) and a conventional stool. The dental material (composite) and dental tools were provided by the researchers. The ergonomic supports used to generate the different conditions were an ergonomic stool with adjustable lumbar support Sit-Up Balance Chair^®^ (MeridentOptergo AB, Mölnlycke, Sweden) and a magnification lenses Ultralight Flip-up Loupes^®^ (MeridentOptergo AB, Mölnlycke, Sweden) These lenses are a novel telemicroscope that provides a magnification of 2.5×, while the tilt of the lens and the frame provides a correct angle of vision without tilting of the head (Figure 1).

Electrical muscle activity of the Upper Trapezius (UT), Lateral Deltoid (LD) and Anterior Deltoid (AD) were recorded during the dental tasks using surface electromyography. A laboratory system, the data-LINK900 software v.7.5 (Biometrics Ltd., Newport, UK), was used. Five active SX230 sEMG sensors (Biometrics Ltd., Newport, UK) were employed for the measurements. sEMG bipolar electrodes were placed on the appropriate locations, using double-sided die-cut tapes provided by the manufacturer. All sEMG signals were amplified and sampled at 1000 Hz. Raw bipolar EMG data were processed by using the root-mean-square (RMS) with 50 ms and 64 Hz filter.

### 2.4. Procedures

The study protocol took place on two days, one week apart. On the first day, demographic and anthropometric characteristics were obtained by self-report. During the same visit, participants received a talk on ergonomics and the use of magnification lenses and the ergonomic stool. In addition, an expert optician individually adapted the prismatic glasses for each dentist, so that dentists could become accustomed to the same during the week prior to the data collection of muscle activity.

On the second day, data collection was divided into three phases: preparation, baseline recordings and experimental recordings. First, the subjects were led to a simulated dental workstation for the application of the sEMG electrodes. The skin over the muscles was cleaned with alcohol. SX230 sensors ware placed parallel to the fibers of the selected muscles with the double-sided adhesive tape following the anatomical locations recommended by SENIAM and Motmans et al. [31]. A ground electrode was placed on the wrist.

In order to normalize the data obtained, three maximum voluntary isometric contractions (MVIC) of each measured muscle were recorded following a muscle testing procedure. The maximum and mean amplitudes of the sEMG obtained during the functional exercises was normalized with the highest value of the MVIC and expressed as a percentage of the MVIC (% MVIC). This procedure required three maximal resisted voluntary contractions, which were performed in the sitting position and in the following order: shoulder flexion for the AD, side flexion of the neck and elevation of the shoulder girdle for the UT, and shoulder abduction for LD. After collecting the maximal contractions, the participants rested for 10 min before the baseline recordings were conducted.

Prior to recording the measurements, the starting position of the dentists at the workstation was standardized as follows: the dentist was sitting next to the dental model enabling an approach on the dominant side, the legs were separated and the feet were flat on the floor, the knees were at 90° flexion, and the angle between the lumbar spine and the femur was 110°, to avoid rectification of lordosis, the back was straight, and shoulders were parallel to the horizontal plane with arms and elbows placed alongside the body. Although all dentists began the procedure from the same starting position, they were allowed to adopt their usual posture once the recording began.

The baseline sEMG recordings were established for each subject while performing the tasks in normal conditions with a conventional stool. The experimental records were established for each of the three different ergonomic conditions (ergonomic stool, magnification lenses and both). Both baseline and experimental EMG measures were averaged for each task, condition and muscle group, and then normalized by their respective MVIC.

### 2.5. Statistical Analysis

IBM SPSS^®^ statistics v.23.0 (IBM, Chicago, IL, USA) was used for statistical analysis. The relative test-retest reliability was evaluated using intraclass correlation coefficients (ICCs), and the absolute reliability was determined using the standard error of measurement (SEM). The SEM is presented in absolute reliability terms and as an SEM% by dividing the SEM by the average of the test and retest values. A repeated measures analysis of variance (ANOVA) was used to determine mean differences in sEMG values for each muscle group between the four ergonomic conditions (conventional stool, ergonomic stool, magnification lenses, ergonomic stool & magnification lenses). In addition, we performed multiple post hoc comparisons tests between ergonomic conditions to determine which conditions were significantly different from others, using the Bonferroni *t*-test. We used a significance level of α < 0.05 for all analyses.

## 3. Results

A total of 36 participants (19 males and 17 females) completed all of the tasks with all the ergonomic supports. Their ages ranged from 20 to 59 (39.5 ± 19.5) years old. Of these, 26 (72.2%) participants were included in the test-retest reliability study. Participants and non-participants in this reliability study did not differ regarding sex (*p* = 0.178 and *p* = 0.425, respectively), age (*p* = 0.953; *p* = 0.310) or education level (*p* = 0.365; *p* = 0.067). 

Table 1 presents the reliability of sEMG measurements for each muscle and task. All measurements showed excellent ICCs, and all confidence intervals had a lower limit that was higher than 0.8. The standard error of measurement varied between 0.7% and 6.4% for the different measurements. The measurement of the UT in the filling task was the only muscle where the measurement error was slightly above 5%. All muscles showed a comparable measurement error between the different tasks. 

Table 2 displays the mean sEMG values (and SD) expressed as a percentage of MVIC for each of the four ergonomic conditions and the three tasks. The results of the one way repeated-measures ANOVA showed that the UT muscle activity differed significantly across the four ergonomic conditions in the three tasks (*F* = 35.82 (*p* < 0.001), *F* = 30.06 (*p* < 0.001), *F* = 28.72 (*p* < 0.001), respectively). Bonferroni post hoc tests showed that the UT had more muscle activity in the condition without ergonomic support compared to each one of the three ergonomic supports (*p* < 0.001) in the three tasks. Moreover, the difference among these three conditions also reached significance (*p* < 0.001). 

**Figure 2:**
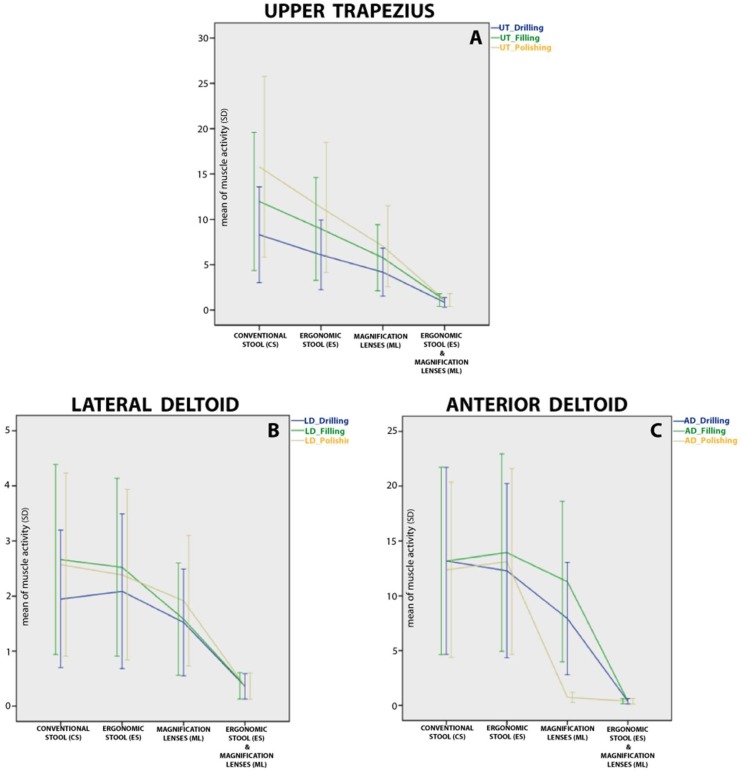
Mean and standard deviation (SD) of muscle activity for upper trapezius (**A**), lateral deltoid (**B**) and anterior deltoid (**C**) in different ergonomic conditions and tasks: drilling, filling and polishing.

**Figure 3:**
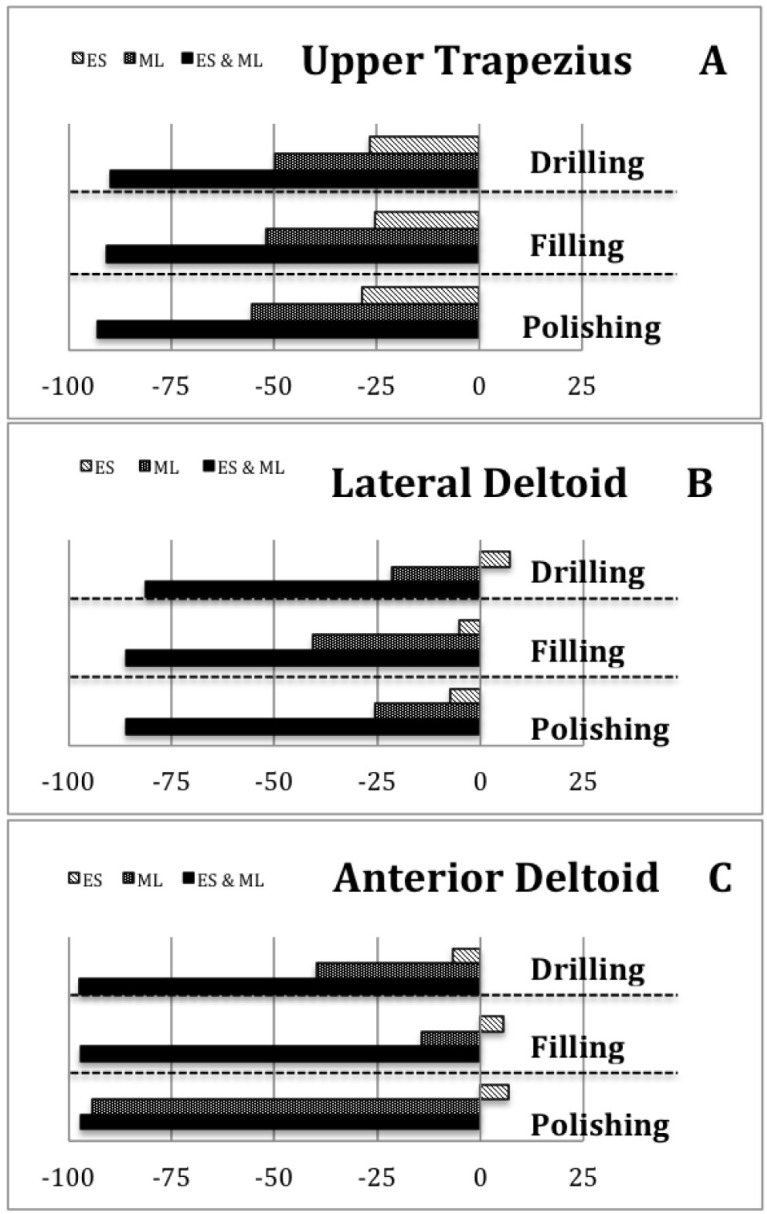
Percentage of change in muscle activity of (**A**) upper trapezius, (**B**) lateral deltoid and (**C**) anterior deltoid (reference: conventional stool) by ergonomic support.

Figure 2A provides a clearer view of the pattern of change across these three conditions (conventional stool, ergonomic stool and magnification lenses), in which the muscle activity decreased in a uniform pattern for the three tasks. Moreover, it also shows how the lowest UT muscle activity occurs when participants use a combination of the ergonomic stool and magnification lenses. Figure 3A shows the percentage of change of the ergonomic supports compared to the conventional stool in order to quantify patterns of change. It also shows how, for the three tasks, the combination of ergonomic stool & magnification lenses reduced UT muscle activity between 89% and 93%. 

Regarding LD and AD, there was also a significant main effect of the ergonomic conditions on the muscle activity of the LD and AD in the three tasks (Table 2). Bonferroni post hoc tests also showed differences between the three ergonomic supports (*p* < 0.001). However, the difference between the conventional stool and the ergonomic stool showed a slightly different pattern for the UT in some tasks (Figure 2B,C). In addition, the muscle activity of the LD using the ergonomic stool was significantly lower than using the conventional stool only for the filling and polishing tasks (2.52 vs. 2.66 (*p* < 0.001) and 2.38 vs. 2.57 (*p* < 0.001), respectively). This muscle did not show differences in the drilling task (2.08 vs. 1.94). Furthermore, while the muscle activity of the AD was significantly lower using ergonomic stool compared to the conventional stool in the drilling task (12.29 vs. 13.18 (*p* < 0.001)), it was significantly higher in the filling and polishing tasks (13.93 vs. 13.17 (*p* < 0.001) and 13.12 vs. 12.37 (*p* < 0.001), respectively). Figure 3B,C quantify all these patterns of change compared to the conventional stool. Furthermore, the combination of ergonomic supports (ergonomic stool and magnification lenses) reduced LD and AD muscle activity during the three tasks by between 81–97%.

## 4. Discussion

According to our results, the use of ergonomic supports such as the magnification lenses, the ergonomic stool or the combination of both is effective for decreasing the muscle activity of the UT, AD and LD during the three tasks performed during posterior composite restoration, compared to standard practice with the conventional stool. In addition, we determined that the decrease of muscle activity is higher using magnification lenses compared to using the ergonomic stool, and the greatest decrease occurs when both are combined. 

To the best of our knowledge, this study is the first to evaluate the muscle activity of UT, LD and AD, comparing the effect of magnification lenses, ergonomic stool or the combination of both. Our sEMG results can only be partially compared with previous studies. In fact, only one study [13] has evaluated muscle activity in relation to the use of any ergonomic support. This former study found that muscle activity of UT decreased (was lower) with the ergonomic stool versus conventional stool. These authors affirmed that this lower muscle activity registered with the ergonomic stool can be attributed to a better posture. Our results regarding the UT activity when using the stool are consistent with Haddad et al. Furthermore, we agree that a better posture can explain our results. Many studies have shown that spinal posture is significantly less flexed when using an ergonomic stool [16].

In our study, UT muscle activity was lower using magnification lenses than an ergonomic stool. A possible explanation for this may be because the magnification lenses enable dentists to remain in a more upright posture, reducing neck flexion [32]. Theoretically, the magnification lenses provide dentists with a better field of vision without the need to bend forwards as much, therefore reducing trunk flexion. Likewise, many studies have found that using magnification lenses leads to significantly better postures compared to postures employed when using traditional safety glasses [33]. However, there are no studies comparing working postures associated to the use of magnification lenses and the ergonomic stool. In our opinion, our study highlights that dentists’ posture may be different using these two ergonomic supports.

We found that the combination of the ergonomic stool and magnification lenses provided superior results for the muscle activity of UT than the use of isolated ergonomic supports. A possible reason for this could be a synergistic positive effect on posture from both ergonomic supports. Thus, the combination of the ergonomic stool and magnification lenses may facilitate a more upright posture compared to an individual support. The fact that the combination of supports generated such low muscle activity in UT, and even regarding other muscles (AD and LD) was an unexpected finding. In our opinion, this surprising effect cannot be attributed to measurement errors because our preliminary study revealed very reliable measures, with a percentage of standard error measure below 5%. Thus, the effect of the ergonomic stool is greater when the dentist wears magnification lenses. This can be explained because of the maintenance of the physiological lumbar lordosis thanks to the ergonomic stool, which is increased when combined with the upright posture generated by magnification lenses.

The muscle activation of AD and LD was similar for the conventional stool and the ergonomic stool however, this was lower for magnification lenses and the combination of magnification lenses and the ergonomic stool. This finding serves to highlight that the use of the ergonomic stool does not modify the dentists’ shoulder flexion/abduction position during dental procedures. Once again, the more upright trunk posture could explain these results. Thus, if the ergonomic stool does not modify hip and trunk flexion enough, dentists need to maintain a similar shoulder flexion/abduction position as when working with the conventional stool. In contrast, when dentists have a more upright position using both ergonomic supports without the need to bend over so much [17], they may require less shoulder flexion/abduction. 

Our reliability study of sEMG measurements during the three tasks confirmed that the sEMG is a reliable tool for the measurement of neck and shoulder muscle activity in dentists. These findings coincide with authors such as Chaikumarn et al. [34], whose study population was office workers with cervical symptoms, and Machado et al. [35], who performed measurements on women with and without neck-shoulder symptoms. We were unable to find reliability studies of the sEMG on dentists.

A limitation of the present study was that sEMG activities of the UT, LD and AD were only collected from the dominant side and no inference can therefore be made regarding the contraction pattern of the muscles investigated on the non-dominant side. Another limitation was that we did not register the changes in the dentists’ position during tasks, as our only outcome measure was muscle activity.

## 5. Conclusions

In summary, our study found that while the use of a magnification lenses is effective for decreasing the muscle activity of all the muscles studied, the use of the ergonomic stool alone reduced the muscle activity of the UT, while yielding a disparate pattern in the AD and LD muscle groups. The best results were obtained with the combination of both ergonomic supports, producing a highly significant reduction in the muscle activity of all muscles compared with the isolated use of these supports.

## Figures and Tables

**Table 1:** Reliability of surface electromyography (sEMG) measurements for each muscle and task.

Muscle	Task	ICC (IC 95%)	SEM	%SEM
UT	Drilling	0.992 (0.982; 0.996)	0.14	2.3
	Filling	0.972 (0.938; 0.987)	0.59	6.4
	Polishing	0.990 (0.977; 0.995)	0.32	2.6
LD	Drilling	0.966 (0.925; 0.984)	0.06	4.6
	Filling	0.992 (0.982; 0.996)	0.04	2.2
	Polishing	0.977 (0.950; 0.990)	0.07	3.8
AD	Drilling	0.994 (0.986; 0.997)	0.27	2.7
	Filling	0.999 (0.998; 1.000)	0.07	0.7
	Polishing	0.977 (0.950; 0.990)	0.33	3.5

UT: Upper Trapezius; LD: Lateral Deltoid; AD: Anterior Deltoid; ICC: Intraclass Correlation Coefficient; SEM: Standard Error of Measurements.

**Table 2:** Mean of muscle activity in different ergonomic conditions and tasks by muscle.

Muscle	Task	CS Mean (SD)	ES Mean (SD)	ML Mean (SD)	ES & ML Mean (SD)	Significance		
						Wilks’ Lambda	*F* Statistic	*p*-Value
UT	D	8.30 (5.28)	6.08 (3.8)	4.17 (2.64)	0.84 (0.53)	0.235	*F*_3.33_ = 35.82	<0.001
	F	11.97 (7.61)	8.94 (5.66)	5.76 (3.64)	1.09 (0.68)	0.268	*F*_3.33_ = 30.06	<0.001
	P	15.80 (9.96)	11.31 (7.17)	7.04 (4.46)	1.10 (0.69)	0.277	*F*_3.33_ = 28.72	<0.001
LD	D	1.94 (1.24)	2.08 (1.40)	1.52 (0.97)	0.36 (0.23)	0.280	*F*_3.33_ = 28.32	<0.001
	F	2.66 (1.72)	2.52 (1.61)	1.58 (1.01)	0.37 (0.24)	0.235	*F*_3.33_ = 35.87	<0.001
	P	2.57 (1.66)	2.38 (1.54)	1.91 (1.18)	0.36 (0.24)	0.225	*F*_3.33_ = 37.88	<0.001
AD	D	13.18 (8.53)	12.29 (7.95)	7.92 (5.13)	0.35 (0.23)	0.284	*F*_3.33_ = 27.68	<0.001
	F	13.17 (8.54)	13.93 (9.01)	11.29 (7.31)	0.36 (0.22)	0.272	*F*_3.33_ = 29.38	<0.001
	P	12.37 (8.00)	13.12 (8.48)	0.71 (0.46)	0.36 (0.24)	0.276	*F*_3.33_ = 28.90	<0.001

UT: Upper Trapezius; LD: Lateral Deltoid; AD: Anterior Deltoid; D: Drilling; F: Filling; P: Polishing; CS: Conventional Stool; ES: Ergonomic Stool; ML: Magnification Lenses.
